# Systematic Differences in Corneal Curvature and Power Measurements Between the IOLMaster 700 and the Anterion and Mapping Strategies for Cross-Device Use

**DOI:** 10.3390/diagnostics16162556

**Published:** 2026-08-13

**Authors:** Achim Langenbucher, Jascha Armin Wendelstein, Alan Cayless, Peter Hoffmann, Nóra Szentmáry

**Affiliations:** 1 Department of Experimental Ophthalmology, Saarland University, 66424 Homburg, Germany; wendelsteinjascha@gmail.com (J.A.W.); nszentmary@gmail.com (N.S.); 2 Department of Ophthalmology, Ludwig-Maximilian University, 80336 Munich, Germany; 3 IROC AG, Institut für Refraktive und Ophthalmo-Chirurgie, 8006 Zurich, Switzerland; 4 School of Physical Sciences, The Open University, Milton Keynes MK7 6AA, UK; alan.cayless@gmail.com; 5 Augen- und Laserklinik Castrop-Rauxel, 44575 Castrop-Rauxel, Germany; pupillenpeter@gmx.de; 6 Department of Ophthalmology, Semmelweis University, Mária u. 39, 1085 Budapest, Hungary

**Keywords:** optical biometry, IOLMaster 700, Anterion, corneal back surface curvature, corneal tomography, total corneal power, power vector analysis, mapping strategies, intraocular lens power calculation, device agreement

## Abstract

**Background/Objectives**: Corneal power has the largest impact on the variability of intraocular lens (IOL) power predictions, and corneal data from different biometers cannot be used interchangeably. We quantified the systematic differences between the Zeiss IOLMaster 700 (IOLM) and the Heidelberg Engineering Anterion and derived strategies for using both devices interchangeably in IOL power calculation. **Methods**: In this retrospective single-centre study, 837 eyes of 837 cataract patients were measured preoperatively with both biometers. Harmonic mean corneal front and back surface radii were derived from the flat and steep meridians, and corneal power referenced to the front apex plane was expressed as spherocylindrical power vectors (spherical equivalent, SEQ; astigmatic components C0 and C45). Three mapping strategies were compared using Bland–Altman and double-angle plots: linear regression of corneal radii without (MR) and with (MRI) intercept and multivariate linear regression of the power vector components (MMV). **Results**: Corneal front surface radii agreed well between devices (MR slope 1.000), whereas the IOLM reported systematically flatter posterior radii (MR slope 0.943), giving a systematically higher total corneal power (43.105 D versus 42.760 D). MRI mapping largely removed the systematic offset in the corneal radii but did not fully correct the astigmatic centroids, whereas MMV mapping aligned both the SEQ and the astigmatic centroids at the origin and yielded smaller confidence ellipses. **Conclusions**: Measurements from the two devices are not directly interchangeable, primarily because of systematic discrepancies in the reported posterior corneal curvature. Where identical IOL calculation concepts and formula constants are used across devices, conversion of corneal data is mandatory, and multivariate power vector mapping provides superior harmonisation compared with radius-based approaches.

## 1. Introduction

When comparing biometric measures performed for lens power calculations before cataract surgery, the mismatch of corneal power measurements as reported by different devices appears to have the largest impact on the variation in intraocular lens (IOL) power predictions or in predictions of the postoperative refraction. Although measures of axial length, central corneal thickness, anterior chamber depth and central crystalline lens thickness are quite consistent between biometers, corneal measures from different biometers cannot be directly compared or used interchangeably [1].

This mismatch in reported corneal power includes corneal front and back surface curvature or power, keratometric power and total power values [2,3,4,5,6,7]. Different devices may use different measurement locations and may also use different calibrations, refractive indices of the ocular media or keratometer indices, or different assumptions regarding the reference plane [2,3]. As a consequence, the corneal measures from different devices cannot be used interchangeably for IOL power calculations. It would be necessary to customise the formula constants for all IOL models to each specific biometer. Alternatively, if it were deemed preferable to use the same set of formula constants across all instruments, it would be necessary to adjust the corneal curvature or power data from each individual biometer accordingly.

In central Europe and in the US, the IOLMaster 700 (IOLM; Carl Zeiss Meditec AG, Jena, Germany, software version 1.90.40.5.C89480) is a widely used optical biometer based on swept-source optical coherence tomography (SS-OCT). This instrument derives corneal front surface curvature using telecentric keratometry measured at 18 corneal locations organised in three radial concentric rings around the corneal apex. The corneal thickness profile is derived using optical coherence tomography, and the IOLM then uses keratometry and the thickness profile to extract the corneal back surface curvature or power in the central 4 to 4.5 mm zone [8]. Over the last ten years, other optical biometers have become more and more popular, some of which are essentially designed as anterior segment tomographers capable of measuring the shape of the corneal front and back surface at thousands of locations [9]. For example, the Anterion (Heidelberg Engineering GmbH, Heidelberg, Germany, software version 1.5.9.0) uses SS-OCT to measure corneal and anterior segment structures with a high resolution, combined with on-axis axial length measurement.

Both instruments provide data on the corneal front and back surface curvature in the flat and steep meridians, together with the axis orientations of both meridians and data on the central corneal thickness. The reliability and repeatability of these instruments has been extensively tested in various studies, and comparisons of both instruments include agreement metrics for corneal curvature as well as biometric measures such as axial length, central corneal thickness or anterior chamber depth [10,11,12,13,14,15,16,17,18,19,20,21]. In addition, both instruments yield measures of the total keratometric power, and the Anterion also reports the total corneal power derived from the corneal front and back surface data and the central corneal thickness [2,11,20]. However, one device shows an anterior to posterior curvature ratio (APR) of 1.19 (Anterion) and the other of 1.12 (IOLM) in the literature.

A further complication is that the “total” results reported by the two instruments are not directly comparable. The IOLM reports a total keratometry (TK) value which refers to the equivalent corneal power referenced to the principal plane of the cornea, whereas the total corneal power values reported by the Anterion are based on a calculation which is not fully disclosed. For this reason, in the present study we define a total corneal power referenced to the front apex plane which can be calculated in the same way for both instruments based on the individual corneal front and back surface curvature and corneal thickness measurements. As most of the formula constants used in IOL power calculations are optimised for the classical IOLM, IOL power calculation with other biometers such as the Anterion requires either customisation of the formula constants or a mapping of the corneal measures to enable formula constants derived from the IOLM to be applied.

The purpose of the present study is therefore threefold:

• To evaluate the differences in corneal curvature and power measures from the corneal front and back surface, comparing the IOLM and the Anterion optical biometer;
• To develop a mapping strategy to statistically convert corneal measurement data from the IOLM to the Anterion and vice versa;
• To investigate the differences between the IOLM and the Anterion with and without mapping in a study population measured before cataract surgery.

## 2. Materials and Methods

### 2.1. Study Population

A large dataset containing 981 biometric measurements (981 eyes of 981 patients) taken prior to cataract surgery was considered in this study. All measurements were performed at the Augen- und Laserklinik, Castrop-Rauxel, Germany, using the IOLMaster 700 and the Anterion biometer. Eyes with any history of ocular surgery or with documented comorbidities in the patient record were excluded from the dataset at source prior to transfer. The data were anonymised at source and transferred to an .xlsx data table using the software module for batch data export. Only one eye per patient was included in the analysis; where data from both eyes were available, a randomly selected eye was chosen, so that inter-eye correlation could not bias the confidence intervals.

Data tables were reduced to the following parameters as required for our analysis: the patient’s age (age) in years, the laterality (left or right eye) and sex (female or male); for the IOLM, the flat (ZR1a) and steep (ZR2a) corneal front surface radii of curvature, both in mm, together with the orientation of the flat axis (ZA1a in degrees) and the steep axis (ZA2a in degrees), the flat (ZR1p) and steep (ZR2p) corneal back surface radii of curvature, both in mm, together with the orientation of the flat axis (ZA1p in degrees) and the steep axis (ZA2p in degrees), and the central corneal thickness (ZCCT in mm); and for the Anterion, the flat (HR1a) and steep (HR2a) corneal front surface radii of curvature, both in mm, together with the orientation of the flat axis (HA1a in degrees) and the steep axis (HA2a in degrees), the flat (HR1p) and steep (HR2p) corneal back surface radii of curvature, both in mm, together with the orientation of the flat axis (HA1p in degrees) and the steep axis (HA2p in degrees), and the central corneal thickness (HCCT in mm). Subjects with missing data, or with data other than ‘OK’ in the internal quality check of the IOLM or the Anterion for any of the above-mentioned parameters, were excluded.

The data were transferred to Matlab (Matlab 2025b; MathWorks, Natick, MA, USA) for further processing.

### 2.2. Preprocessing of the Corneal Data

The mean radii for the corneal front surface (ZRa and HRa, both in mm) and the corneal back surface (ZRp and HRp, both in mm) were calculated as the harmonic mean of the corresponding flat and steep radii for the IOLM and the Anterion. Based on the refractive indices for the cornea (nC = 1.376) and the aqueous humour (nA = 1.336), and with a keratometer index (nK = 1.332), we converted the corneal front and back surface curvature to spherocylindrical vergence vectors (IVj, each with 8 vector elements) representing the front surface power, the keratometric power and the back surface power, defined as follows:

1: IP1j = Δn/IRj, dioptric power in the flat meridian;
2: IA1j, axis of the flat meridian;
3: IP2j = Δn/IRj, dioptric power in the steep meridian;
4: IA2j, axis of the steep meridian;
5: IASTj = IP2j − IP1j, astigmatism;
6: ISEQj = 0.5·(IP1j + IP2j), spherical equivalent power;
7: IC0j = (IP2j − IP1j)·cos(2·IA1j), projection of the astigmatism onto the 0/90 degree meridian;
8: IC45j = (IP2j − IP1j)·sin(2·IA1j), projection of the astigmatism onto the 45/135 degree meridian.

In the labels for each element, ‘I’ refers to the biometer (either ‘Z’ for the IOLM or ‘H’ for the Anterion), and ‘j’ refers to either ‘a’, ‘p’ or ‘k’ to represent the vergence vector for the corneal front surface power (Δn = nC − 1), the back surface power (Δn = nA − nC), or the keratometric power (Δn = nK − 1), respectively. Algebraic operations for the vergence vectors include vergence addition (ADD, where elements 6, 7 and 8 of the respective vergences are added up and elements 1 to 5 are updated) and vergence transfer through a homogeneous optical medium (TRANS, where elements 1 and 3 are transferred with vergence transformation, elements 2 and 4 are kept unchanged, and elements 5 to 8 are updated).

### 2.3. Mapping from the IOLM to the Anterion and from the Anterion to the IOLM

Various mapping strategies for converting corneal data from the IOLM to the Anterion and from the Anterion to the IOLM were tested. First, we used a simple scaling model (MR, linear regression without intercept) to convert the mean corneal front and back surface radii from the IOLM to the Anterion and vice versa. In the next step we used a linear regression model with intercept (MRI) to map the mean corneal front and back surface radii from the IOLM to the Anterion and vice versa. Finally, we used a multivariate linear model (MMV) [4,8,17] to map the 3 power vector components (SEQ, C0 and C45) for the corneal front and back surface from the IOLM to the Anterion and from the Anterion to the IOLM.

Using the conversion models for the mean corneal radii (MR and MRI) we calculated the corrected corneal front surface radii for the flat and steep meridians for the IOLM which give the best match to the Anterion and those for the Anterion giving the best match to the IOLM. Using the multivariate model (MMV) we calculated the power vector components SEQ, C0 and C45 for the IOLM which give the best match to the corresponding power vector components of the Anterion and, similarly, those for the Anterion which give the best match to the corresponding power vector components of the IOLM.

### 2.4. Total Corneal Power Data

Total corneal power was derived from the vergence vectors for the corneal front and back surface and the corneal thickness. Total corneal power was referenced to the front apex plane so as to be processed later with IOL power formulae. The vergence vector for the front surface (ZVa for the IOLM or HVa for the Anterion) was transformed to the back surface (TRANS with an optical distance ZCCT/nC for the IOLM or HCCT/nC for the Anterion), and we added the vergence vector for the back surface (ADD ZVp for the IOLM or HVp for the Anterion) to obtain the spherocylindrical back vertex power of the cornea. The total corneal power referenced to the front apex plane was extracted by transforming the back vertex power back to the front apex plane (TRANS with an optical distance −ZCCT/nA for the IOLM or −HCCT/nA for the Anterion).

In order to provide a common ground for comparison, we decided to calculate total corneal power values for both biometers in the same way, based on the corneal front and back surface curvature and the corneal thickness [8,17]. This was necessary since the total power values as reported by the two instruments are not directly comparable: the built-in total power values (total keratometry, TK) reported by the IOLM refer to the equivalent corneal power referenced to the principal plane of the cornea, whereas the total corneal power values reported by the Anterion are not fully disclosed (the user manual does not specify the reference plane).

### 2.5. Statistical Analysis and Data Presentation

Explorative data for the measured corneal front and back surface radii, the corneal front and back surface radii corrected with the MR and MRI models, the power vector components for the measured corneal front and back surface, and the power vector components corrected with the MMV models are listed in terms of the arithmetic mean, standard deviation (SD), median, and the lower and upper boundary of the 95% confidence interval. For the MR and MRI models we list the coefficients (slope and slope and intercept) together with their 95% confidence intervals, the root-mean-squared (RMS) model fit error, and the coefficients of determination (R^2^). For the MMV models we list the RMS model fit error and R^2^. The differences in the mean corneal front and back surface radii measured with the IOLM (ZRa and ZRp) and the Anterion (HRa and HRp) are reported using Bland–Altman plots, together with the respective corrections using the MRI models. The power vector components for the total corneal power derived from the measured corneal front and back surface data, as well as those obtained after correction with the MMV models, are displayed using combined violin plots for the SEQ components and double-angle plots for the C0 and C45 astigmatic power vector components.

## 3. Results

### 3.1. Study Population and Descriptive Data

Of the N = 981 measurements from the IOLM and the Anterion biometer transferred to us, a total of N = 837 were used after eliminating measurements with incomplete data or with a quality check other than ‘OK’. The mean patient age at the time of examination was 72.33 ± 10.13 years (median 72.82 years, 95% confidence interval from 52.79 to 88.27 years), and 400 left and 437 right eyes were considered. Table 1 shows the descriptive data for the measured corneal front and back surface radii together with the central corneal thickness, and Table 2 displays the descriptive data for the corneal front surface, corneal back surface and keratometric power vector components derived from the corneal radii for the IOLM and the Anterion.

### 3.2. Mapping Models

Table 3 shows a listing of the model parameters mapping the mean corneal front and back surface radii from the IOLM to the Anterion (middle column) or from the Anterion to the IOLM (right column). The upper part of the table lists the parameters characterising the linear MR models without intercept, the middle part details the parameters of the linear MRI models with intercept, and the lower part gives the parameters of the multivariate models, which map the power vector components for the front and back surface from one biometer to another. It is clear from the table that the corneal front surface radii match well between the IOLM and the Anterion, with the slopes of the MR models close to 1. However, we note from the MRI model mapping ZRa to HRa that the regression line shows a noticeable intercept of around 0.186 mm and as a consequence a slope lower than 1 (0.976). For the corneal back surface radii we see that there is a systematic mismatch between the two biometers: the IOLM consistently overestimates the corneal back surface radii of the Anterion (slope of the MR model 0.943), or conversely the Anterion systematically underestimates the corneal back surface radii of the IOLM (slope of the MR model 1.060).

### 3.3. Agreement of the Corneal Radii

**Figure 1:**
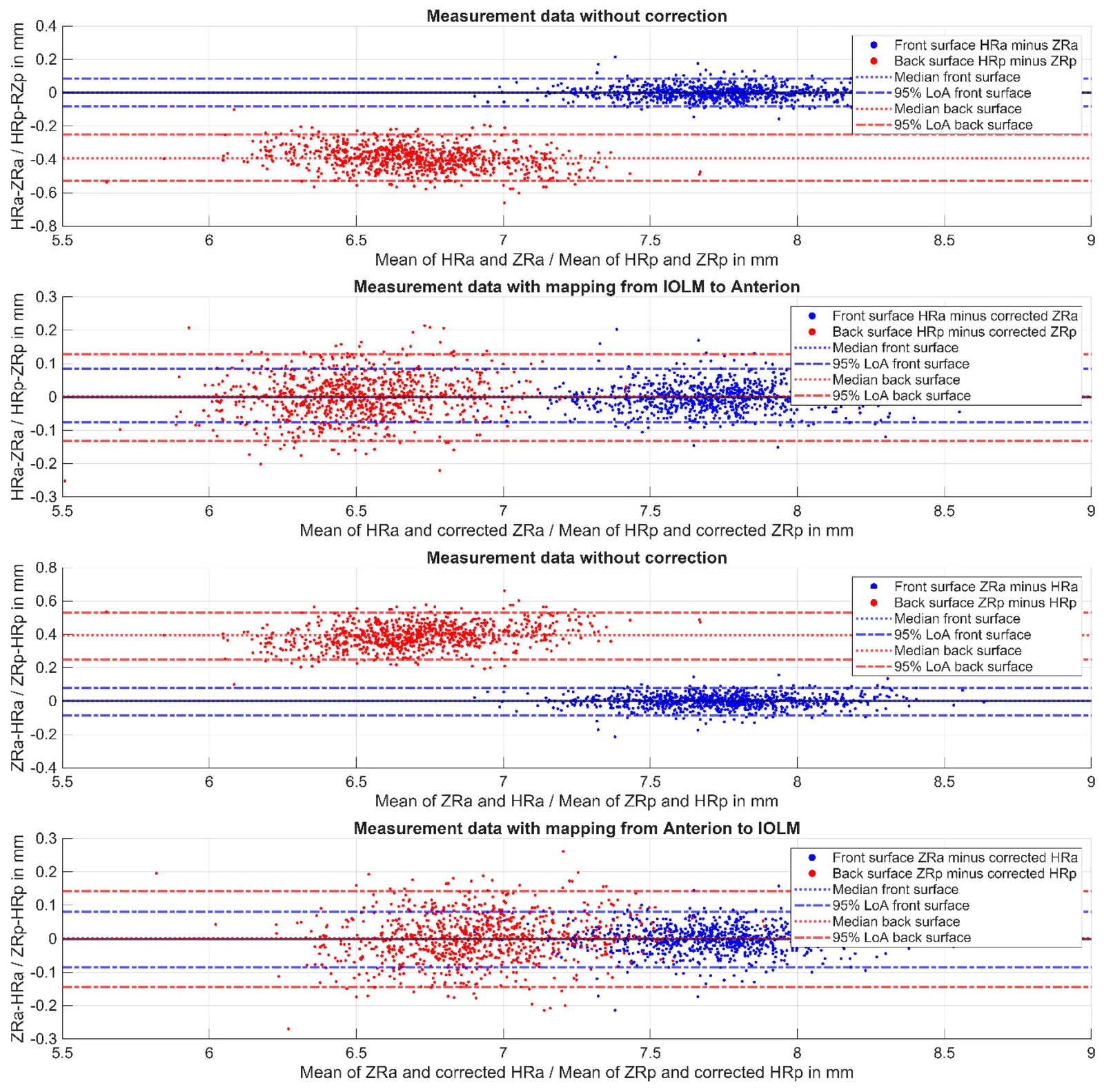
Bland–Altman plots comparing the mean corneal front and back surface radii for the Zeiss IOLMaster 700 (IOLM) and the Heidelberg Engineering Anterion (Anterion). The first plot shows the difference in the Anterion minus the IOLM data and the second plot the differences after mapping from the IOLM to the Anterion using the linear regression model with intercept (MRI). The third plot shows the difference in the IOLM minus the Anterion data and the fourth plot the differences after mapping from the Anterion to the IOLM using the MRI model. The horizontal lines indicate the median and the lower and upper boundaries of the 95% limits of agreement.

Figure 1 shows the Bland–Altman plots comparing the corneal front and back surface radii of the IOLM and the Anterion for the measurement data (first plot: Anterion minus IOLM; third plot: IOLM minus Anterion) together with the corneal front and back surface radii mapped with the MRI models from the Anterion to the IOLM (second plot) and from the IOLM to the Anterion (fourth plot) as specified in Table 3. We clearly see from the first and third plots that the corneal front surface radii of both biometers match well, whereas the IOLM/Anterion systematically overestimates/underestimates the corneal back surface radius. After correction with the MRI models we still observe some stochastic scatter, but the systematic mismatch, especially of the corneal back surface, is largely eliminated. In general, the plots indicate that the differences between both biometers are systematically larger for the corneal back surface compared with the corneal front surface.

### 3.4. Total Corneal Power and the Effect of Mapping

**Figure 2:**
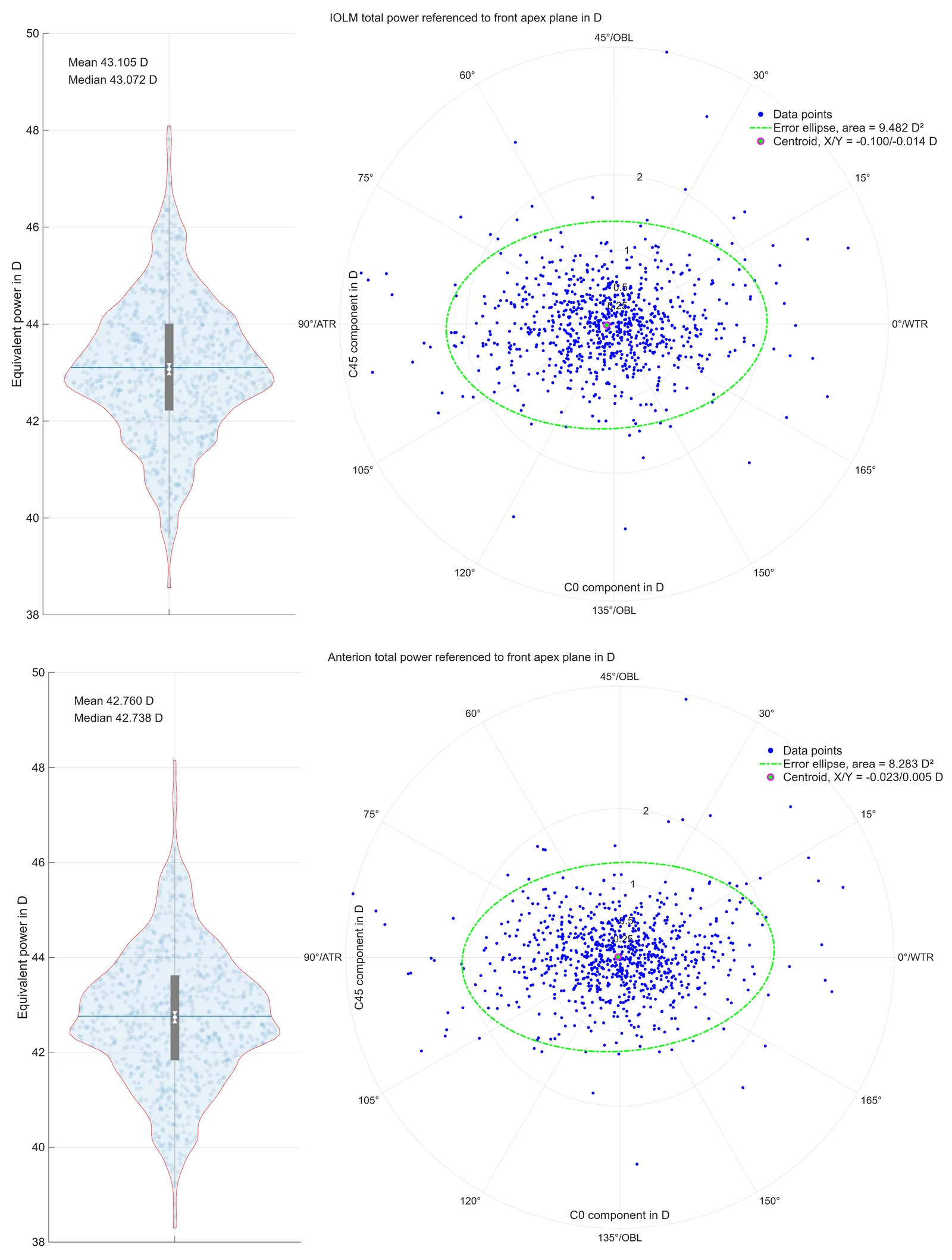
Distributions of the power vector components for the total corneal power (referenced to the corneal front apex plane) of the Zeiss IOLMaster 700 (upper graphs) and the Heidelberg Engineering Anterion (lower graphs). The violin plots on the left side indicate the distributions of the spherical equivalent power with the median (blue horizontal line; median and mean given in the legend) and the interquartile range (grey bar). The double-angle plot on the right side shows the astigmatic power vector components (C0 in X and C45 in Y) together with the centroid and the 95% confidence ellipse (centroid location and ellipse area given in the legend).

Figure 2 displays the distributions of the power vector components for the total corneal power referenced to the corneal front apex plane, as derived from the measurement data from the IOLM (upper graph) and from the Anterion (lower graph). The left side of the plot shows the distributions of the SEQ power vector components as a violin plot, and on the right side is a double-angle plot for the astigmatic power vector components C0 and C45. Comparing the SEQ distributions of the total corneal power between the IOLM and the Anterion, we see that the IOLM (mean 43.105 D) provides a systematically higher power compared with the Anterion (mean 42.760 D). From the double-angle plots we can see, from the horizontal shape of the error ellipse, that the astigmatism at 0 and 90 degrees (with-the-rule and against-the-rule) is systematically more pronounced compared with the astigmatism in the oblique axes (oblique astigmatism). The centroid location for the IOLM is slightly shifted to the left, indicating a more pronounced astigmatism against-the-rule compared with the Anterion.

**Figure 3:**
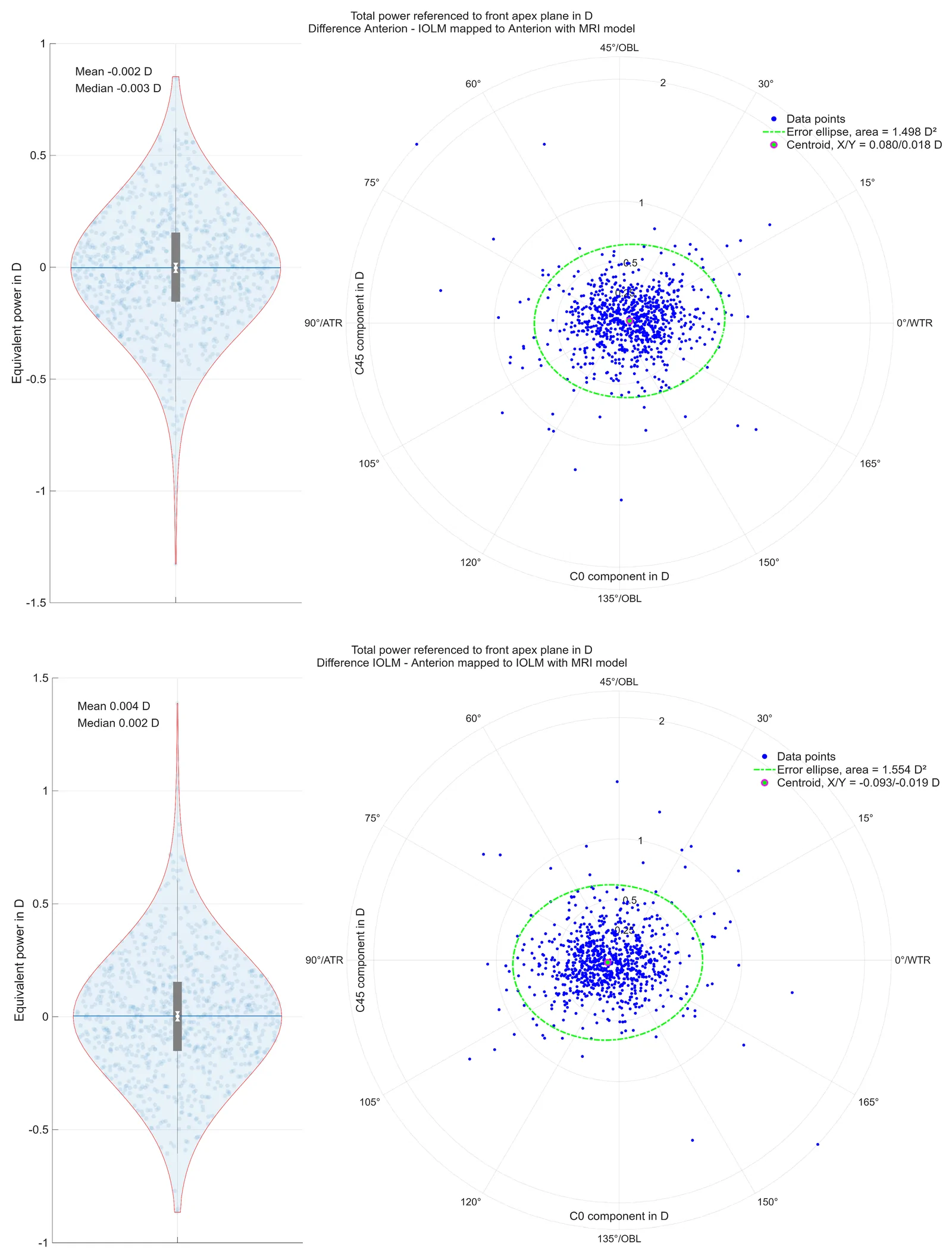
Distributions of the differences in the power vector components for the total corneal power (referenced to the corneal front apex plane) of the Heidelberg Engineering Anterion minus the Zeiss IOLMaster 700 mapped to the Anterion using the linear MRI model (upper graphs), and of the IOLM minus the Anterion mapped to the IOLM using the linear MRI model (lower graphs). The specifications of the MRI models are listed in Table 3. The violin plots on the left side indicate the distributions of the spherical equivalent power with the median (blue horizontal line; median and mean given in the legend) and the interquartile range (grey bar). The double-angle plot on the right side shows the astigmatic power vector components (C0 in X and C45 in Y) together with the centroid and the 95% confidence ellipse (centroid location and ellipse area given in the legend).

Figure 3 shows the distributions of the power vector component differences for the total corneal power (referenced to the corneal front apex plane) as derived from the measurement data from the Anterion minus the IOLM mapped to the Anterion (upper graph) and the IOLM minus the Anterion mapped to the IOLM (lower graph) using the linear MRI model with intercept as specified in Table 3. Even though the distributions for the SEQ differences range around zero (mean −0.002 D for the upper and 0.004 D for the lower graph), the MRI model does not fully correct for the centroid error of the astigmatic power vector components as shown in the double-angle plots on the right side. The centroid in the upper graph is located to the right of the origin (X = 0.080 D), indicating on average a slight trend towards astigmatism with-the-rule with the Anterion, and the centroid in the lower graph is located to the left of the origin (X = −0.093 D), indicating on average a slight trend towards astigmatism against-the-rule with the IOLM.

**Figure 4:**
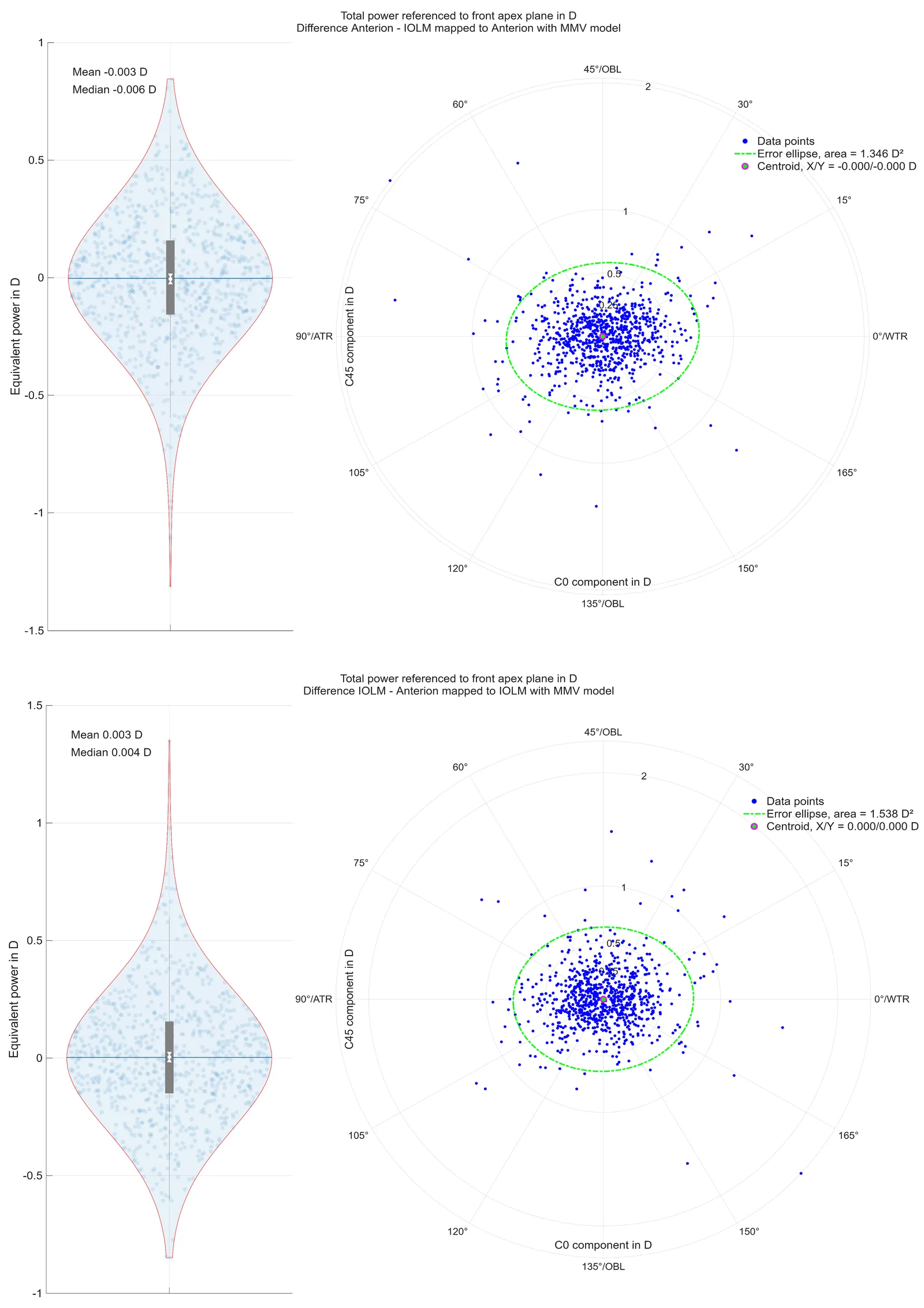
Distributions of the differences in the power vector components for the total corneal power (referenced to the corneal front apex plane) of the Heidelberg Engineering Anterion minus the Zeiss IOLMaster 700 mapped to the Anterion using the multivariate linear MMV model (upper graphs), and of the IOLM minus the Anterion mapped to the IOLM using the multivariate linear MMV model (lower graphs). The specifications of the MMV models are listed in Table 3. The violin plots on the left side indicate the distributions of the spherical equivalent power with the median (blue horizontal line; median and mean given in the legend) and the interquartile range (grey bar). The double-angle plot on the right side shows the astigmatic power vector components (C0 in X and C45 in Y) together with the centroid and the 95% confidence ellipse (centroid location and ellipse area given in the legend).

Figure 4 shows the distributions of the power vector component differences for the total corneal power (referenced to the corneal front apex plane) as derived from the measurement data from the Anterion minus the IOLM mapped to the Anterion (upper graph) and the IOLM minus the Anterion mapped to the IOLM (lower graph) using the multivariate linear MMV model. Again, the distributions for the SEQ differences range around zero (mean −0.003 D for the upper and 0.003 D for the lower graph). With the MMV models, the centroid error of the astigmatic power vector components as shown in the double-angle plots on the right side appears to be fully corrected. Both centroids (in the upper and lower graph) are located exactly at the origin, and the performance of the mapping seems to be slightly better, as indicated by the smaller area of the error ellipses mentioned in the legends of the double-angle plots.

## 4. Discussion

It is generally accepted in ophthalmology that corneal power measures are among the most critical parameters in IOL power calculation [1,8,22]. In daily clinical practice the ‘real’ corneal curvature or power does not play a role; only the changes over time or variations in some specific corneal aberrations might be relevant [2,3]. This means that as long as we use the same instrument or instruments with the same calibrations, we are on the safe side [3].

However, for IOL power calculation we do require reliable measures for the ‘real’ corneal curvature or power [1]. Several modern optical biometers are capable of measuring the back surface curvature of the cornea and the corneal thickness, in addition to the corneal front surface curvature. With these additional data it is possible to derive the total corneal power, which makes the measurement relatively independent of a corneal model required for keratometry. This means that with corneal back surface measurements we do not need any assumptions regarding the anterior to posterior curvature ratio or the corneal thickness. However, we should be careful with the interpretation of total corneal power data, and we have to double-check whether identical refractive indices are used for the conversion from radii to power and whether the total power data are indexed to the same reference plane [2,3].

In reality, corneal back surface measurement is quite difficult, as the measurement is always affected by the refraction of the corneal front surface. As shown in the present study, corneal front surface curvature measures are quite consistent on average, whereas corneal back surface curvature shows systematic differences between devices. For instance, if we use a simple linear model without intercept, we find that the IOLM measures the corneal back surface around 6% flatter compared with the Anterion. As a consequence, the (negative) corneal back surface power is underestimated and, based on the same refractive indices and reference plane, we obtain a higher total corneal power for the IOLM [8,17].

In general, without strict conventions, corneal power metrics are quite inconsistent in the literature. The term total corneal power [23] is not well-defined, and it could refer to the equivalent power, which is referenced to the image-side principal plane of the cornea (typically located some 50 microns in front of the cornea), to the back vertex power (referenced to the corneal back vertex plane), or it could be referenced to the corneal front vertex plane. With identical measures for the corneal front and back surface and corneal thickness, the equivalent power typically gives the lowest value, followed by the power referenced to the corneal front apex plane, whereas the back vertex power gives the largest power value. Since biometers set their coordinate origin to the corneal front apex, it is logical to use a simplified optical model for the IOL power calculation in which the thin replacement lens for the cornea is located at the corneal front apex plane. This means that the term ‘total corneal power’ should refer to the power of the cornea referenced to the front apex [8,17]. The same applies to the keratometric power and total keratometric power concepts.

In the present study we used a large dataset with biometric measurements taken before cataract surgery, in which measures from both the IOLM and the Anterion were available. Corneal front and back surface curvature in the flat and steep meridians were extracted together with the corneal thickness. To be consistent with the equivalent power of the corneal front and back surface, we used the harmonic mean to derive the mean corneal front and back surface radii. If we compare corneal radii between the IOLM and the Anterion we clearly see from Figure 1 and Table 1 that ZRa and HRa match quite well, whereas ZRp systematically exceeds HRp. Using the corneal radii of the IOLM and the Anterion interchangeably (i.e., in the same IOL power calculation formula with the same formula constants) requires a mapping of the corneal measures from one device to the other.

The simplest setting for such a mapping is a linear regression without intercept (scaling), and our MR model indicates that the scaling factors to transfer ZRa to HRa or HRa to ZRa are both 1.00, whereas the scaling factor to transfer ZRp to HRp is 0.94 and that to transfer HRp to ZRp is 1.06. If we use a linear model MRI with intercept instead, we find that the regression models show a slightly better performance (lower RMS model fit error and higher R^2^), but the regression lines show some intercept (e.g., HRp = 0.37 + 0.89·ZRp). If we apply, for example, the MRI mapping model to the corneal front and back surface radii, we see from the second and fourth plots in Figure 1 that the systematic offset between both biometers, especially for the corneal back surface, can be eliminated. However, the larger limits of agreement in the Bland–Altman plots indicate that the corneal back surface measures are less coherent between both biometers compared with the front surface measures.

In addition, we used multivariate linear models (MMV) to map the three power vector components for the corneal front and back surface from the IOLM to the Anterion or from the Anterion to the IOLM. This transfer requires a 3-element intercept vector B and a 3 × 3 scaling matrix A, as shown in Table 3. Comparing the coefficients of determination for the MMV models with those of the MR or the MRI models, the MMV models seem to perform slightly better.

We then calculated the corneal power vectors for the total corneal power referenced to the corneal front apex plane from the original corneal front and back surface radii measurements, from the measured front and back surface radii after applying the MRI mapping, and also from the measured front and back surface radii transferred to power vector components with the MMV models applied. The corresponding power vector components for the total corneal power derived with the IOLM and the Anterion are shown in Figure 2. First, we notice that the spherical equivalent derived with the Anterion is systematically lower compared with the IOLM. This might be mostly due to the flatter corneal back surface measures with the IOLM. In addition, we see that the data scatter of the astigmatic power vector components is slightly larger with the IOLM (area 9.48 D^2^) compared with the Anterion (area 8.28 D^2^), but for both biometers the C0 power vector component is more pronounced than the C45 power vector component (horizontal orientation of the error ellipse).

If we compare the power vector components for the total corneal power referenced to the front apex plane for the IOLM measurements with the Anterion corrected with the MRI model, or the Anterion measurements with the IOLM corrected with the MRI model, we find that on average the spherical equivalent power differences range around zero, but the distributions seem to be somewhat skewed, as shown in Figure 3. For the differences in the astigmatic power vector components we see that the differences in the C0 and C45 components are in the same range (mostly spherical shape of the error ellipses), but the centroids show some horizontal displacement from the origin. This indicates that the linear MRI models with intercept, as a rather simple method to map corneal radii from one biometer to another, do not fully correct for the centroid error in the astigmatic power vector components.

The second option to compensate for the differences between the two devices is to abandon a common set of formula constants and instead use constants that have been optimised specifically for each biometer. Because the Anterion reports systematically steeper posterior corneal radii and, consequently, a lower total corneal power than the IOLM, applying IOLM-optimised constants (for example, those published on the IOLCon platform, https://iolcon.org) to Anterion measurements would introduce a systematic error and should be avoided. Where the required case numbers are available, the cleanest solution is therefore to build a dedicated constant database for the Anterion as the more recent device, so that device-specific constants are used throughout. Constant optimisation and corneal-data mapping are complementary rather than mutually exclusive: mapping offers an immediate route to interchangeability when device-specific constants are not yet available for a given IOL model.

To put these differences into a clinical context: the mean total corneal power referenced to the front apex plane differed by approximately 0.35 D between the two devices (43.105 D for the IOLM versus 42.760 D for the Anterion). As a rule of thumb, an error of this size in corneal power translates into a comparable error in the predicted IOL power at the spectacle plane of roughly 0.2 to 0.3 D if the corneal data are used interchangeably without conversion and with the same formula constants. For the astigmatic components, the residual centroid offsets that remained after radius-based MRI mapping were of the order of 0.08 to 0.09 D, whereas MMV mapping reduced these to essentially zero. While a single such increment may appear small, systematic offsets of this magnitude act in the same direction for every eye and would therefore bias refractive outcomes across a whole cohort, which is precisely the situation that constant optimisation or corneal-data mapping is intended to prevent.

In contrast, if we compare the power vector components for the total corneal power referenced to the front apex plane for the IOLM measurements with the Anterion corrected with the MMV model, or the Anterion measurements with the IOLM corrected with the MMV model, we find that the spherical equivalent power differences still show some skewness in the distributions, as shown in Figure 4. However, for the differences in the astigmatic power vector components we can see that the differences in the C0 and C45 components are again in the same range (mostly spherical shape of the error ellipses) and that the centroids are located at the origin. This indicates that the MMV models are a suitable method to map power vector components for the front and back surface measures from one biometer to another and that they fully correct for the centroid error in the astigmatic power vector components.

### Limitations

This study has some limitations. Firstly, to keep the calculations simple, we restricted the study to three different mapping techniques: a linear model without intercept to map corneal radii, a linear model with intercept to map corneal radii, and a multivariate linear model to map power vector components from one biometer to another. There might be more advanced (e.g., artificial intelligence-based) models which show superior performance, but implementation in consumer software might be challenging. Secondly, to make the results of both instruments comparable, we restricted the comparison to total corneal power values which were referenced to the corneal front apex plane. These total corneal power values were derived from corneal front and back surface measurements as well as from the measurements after applying the mapping models for corneal radii (MR and MRI) or for power vector components (MMV). Thirdly, the study is based on data from a single centre, and both the mapping coefficients and their transferability to other populations or to other device units should be confirmed in independent datasets. In addition, the mapping models (MR, MRI and MMV) were fitted and evaluated on the same dataset, without a split-sample or k-fold cross-validation. Given the large sample size (N = 837) and the small number of free parameters in each model (one or two coefficients for the MR and MRI models, and a 3 × 3 scaling matrix with a three-element intercept for the MMV models), the risk of overfitting is low, and the reported RMS fit errors and coefficients of determination are expected to be close to their out-of-sample values. Nevertheless, a formal split-sample or cross-validation, and ideally external validation on data from other centres and device units, would further strengthen confidence in the specific conversion coefficients reported in Table 3, and we recommend this for future work.

## 5. Conclusions

Corneal curvature and power data derived with the Zeiss IOLMaster 700 and the Heidelberg Engineering Anterion show systematic differences. For the corneal front surface the data of both biometers are quite consistent, whereas for the corneal back surface there are systematic differences, and a transformation of the corneal measures is mandatory before these data from the IOLM and the Anterion can be used interchangeably. Transformation of corneal data could be performed by mapping the corneal front and back surface radii, or alternatively by mapping the power vector components. In particular, where the corneal measures are to be used for lens power calculation with identical calculation concepts and formula constants, clinicians should apply a transformation of corneal data from the IOLM to the Anterion or from the Anterion to the IOLM, by mapping either the corneal front and back surface radii or the corneal front and back surface power vector components.

## Figures and Tables

**Table 1:** Explorative listing of the measured corneal front and back surface radii and central corneal thickness for the Zeiss IOLMaster 700 (IOLM) and the Heidelberg Engineering Anterion (Anterion), with arithmetic mean, standard deviation (SD), median, and the lower and upper boundary of the 95% confidence interval (2.5% and 97.5% quantile). Corneal radius data are listed for the flat meridian, the steep meridian and the harmonic mean.

Data in mm	IOLM Flat	IOLM Steep	IOLM Mean	Anterion Flat	Anterion Steep	Anterion Mean
**Front surface**	**ZR1a**	**ZR2a**	**ZRa**	**HR1a**	**HR2a**	**HRa**
Mean	7.8016	7.6518	7.7256	7.7990	7.6550	7.7261
SD	0.2599	0.2679	0.2592	0.2569	0.2641	0.2562
Median	7.7968	7.6398	7.7148	7.7900	7.6500	7.7147
2.5% quantile	7.2911	7.1295	7.2283	7.3042	7.1408	7.2307
97.5% quantile	8.3366	8.2866	8.2728	8.3200	8.1957	8.2499
**Back surface**	**ZR1p**	**ZR2p**	**ZRp**	**HR1p**	**HR2p**	**HRp**
Mean	7.0564	6.7437	6.8958	6.6511	6.3641	6.5039
SD	0.2774	0.2835	0.2728	0.2557	0.2605	0.2515
Median	7.0511	6.7354	6.8823	6.6500	6.3500	6.4972
2.5% quantile	6.5204	6.2181	6.3692	6.1700	5.8700	6.0251
97.5% quantile	7.6375	7.3087	7.4668	7.1700	6.8600	7.0108
**Corneal thickness**	**ZCCT**			**HCCT**		
Mean	0.5531			0.5445		
SD	0.0452			0.0470		
Median	0.5540			0.5450		
2.5% quantile	0.4788			0.4720		
97.5% quantile	0.6266			0.6146		

**Table 2:** Explorative listing of the dioptric (D) power vector components for the Zeiss IOLMaster 700 (IOLM) and the Heidelberg Engineering Anterion (Anterion) derived from the corneal front and back surface radii, with arithmetic mean, standard deviation (SD), median, and the lower and upper boundary of the 95% confidence interval (2.5% and 97.5% quantile). SEQ refers to the spherical equivalent power, and C0 and C45 to the astigmatic power components projected onto the 0/90 degree and 45/135 degree meridians. The upper and middle data blocks refer to the corneal front and back surface power, and the lower block to the keratometric power.

Data in D	IOLM	IOLM	IOLM	Anterion	Anterion	Anterion
**Front surface**	**ZSEQa**	**ZC0a**	**ZC45a**	**HSEQa**	**HC0a**	**HC45a**
Mean	48.7239	0.1249	−0.0876	48.7200	0.2158	0.0065
SD	1.6338	0.9694	0.6191	1.6169	0.9203	0.5744
Median	48.7372	0.1517	−0.0265	48.7379	0.2390	0.0000
2.5% quantile	45.4501	−1.9380	−1.1790	45.5765	−1.7287	−1.1457
97.5% quantile	52.0175	2.1241	1.2335	52.0007	2.0475	1.1267
**Back surface**	**ZSEQp**	**ZC0p**	**ZC45p**	**HSEQp**	**HC0p**	**HC45p**
Mean	−5.8097	−0.2338	−0.0863	−6.1594	−0.2486	−0.0018
SD	0.2297	0.1350	0.1027	0.2387	0.1333	0.0907
Median	−5.8120	−0.2337	−0.0046	−6.1565	−0.2524	0.0000
2.5% quantile	−6.2802	−0.4746	−0.2866	−6.6389	−0.4770	−0.1850
97.5% quantile	−5.3570	0.0535	0.1971	−5.7055	0.0213	0.1720
**Keratometric**	**ZSEQk**	**ZC0k**	**ZC45k**	**HSEQk**	**HC0k**	**HC45k**
Mean	43.0222	0.1103	−0.0067	43.0187	0.1986	0.0857
SD	1.4426	0.8568	0.5466	1.4277	0.8126	0.5072
Median	43.0339	0.1348	−0.0234	43.0345	0.2111	0.0000
2.5% quantile	40.1315	−1.7112	−1.0408	40.2431	−1.5193	−1.0116
97.5% quantile	45.9303	1.8755	1.0891	45.9155	1.8079	0.9948

**Table 3:** Linear model description for mapping corneal front and back surface data from the Zeiss IOLMaster 700 (IOLM) to the Heidelberg Engineering Anterion (Anterion) (middle column) and from the Anterion to the IOLM (right column). The upper part of the table shows the model parameters for the linear models without intercept (MR) and the linear models with intercept (MRI) mapping the corneal front surface radii (indexed by ‘a’) and back surface radii (indexed by ‘p’) from one biometer to another. The lower part describes the multivariate linear model (MMV) mapping the front and back surface power vector components (spherical equivalent power SEQ and astigmatic power vector components C0 and C45) from one machine to another. For the MR and MRI models we also provide the 95% confidence intervals for the slope and intercept, and for all models we include the root-mean-squared model fit error (RMS) and the coefficient of determination (R^2^).

	Mapping from IOLM to Anterion	Mapping from Anterion to IOLM
Linear model without intercept (MR)	HRa~ZRa Slope: 1.000 [0.9997 to 1.0000] RMS: 0.0489 mm R^2^: 0.9745	ZRa~HRa Slope: 0.9999 [0.9996 to 1.0000] RMS: 0.0409 mm R^2^: 0.9751
	HRp~ZRp Slope: 0.9431 [0.9424 to 0.9437] RMS: 0.0677 mm R^2^: 0.9274	ZRp~HRp Slope: 1.060 [1.059 to 1.061] RMS: 0.0718 mm R^2^: 0.9307
Linear model with intercept (MRI)	HRa~1+ZRa Slope: 0.9760 [0.9654 to 0.9866] Intercept: 0.1856 [0.1037 to 0.2675] mm RMS: 0.0405 mm R^2^: 0.9750	ZRa~1+HRa Slope: 0.9990 [0.9882 to 1.0100] Intercept:0.0072 [−0.0767 to 0.0918] mm RMS: 0.0409 mm R^2^: 0.9751
	HRp~1+ZRp Slope: 0.8894 [0.8729 to 0.9059] Intercept: 0.3708 [0.2571 to 0.4844] mm RMS: 0.0662 mm R^2^: 0.9388	ZRp~1+HRp Slope: 1.0470 [1.027 to 1.066] Intercept: 0.0889 [−0.0372 to 0.2150] mm RMS: 0.0718 mm R^2^: 0.9308
Multivariate linear model (MMV)	$\left[\begin{array}{c} HSEQa\\ HC0a\\ HC45a \end{array}\right]=A\cdot \left[\begin{array}{c} ZSEQa\\ ZC0a\\ ZC45a \end{array}\right]+B$ $A=\left[\begin{array}{ccc} 0.9788 & -0.0217 & 0.0036\\ -0.0041 & 0.8927 & 0.0212\\ -0.0044 & 0.0125 & 0.8333 \end{array}\right]$ $B=\left[\begin{array}{c} 0.9104\\ 0.2706\\ 0.1991 \end{array}\right]$ RMS: 0.2428 D R^2^: 0.9999	$\left[\begin{array}{c} ZSEQa\\ ZC0a\\ ZC45a \end{array}\right]=A\cdot \left[\begin{array}{c} HSEQa\\ HC0a\\ HC45a \end{array}\right]+B$ $A=\left[\begin{array}{ccc} 0.9967 & 0.0173 & -0.0114\\ 0.0093 & 0.9898 & -0.0303\\ 0.0054 & -0.0135 & 0.9697 \end{array}\right]$ $B=\left[\begin{array}{c} 0.1437\\ -0.4793\\ -0.2437 \end{array}\right]$ RMS: 0.2544 D R^2^: 0.9998
	$\left[\begin{array}{c} HSEQp\\ HC0p\\ HC45p \end{array}\right]=A\cdot \left[\begin{array}{c} ZSEQp\\ ZC0p\\ ZC45p \end{array}\right]+B$ $A=\left[\begin{array}{ccc} 1.0851 & -0.0280 & 0.0468\\ 0.0005 & 0.8491 & 0.0672\\ 0.0260 & 0.0173 & 0.6513 \end{array}\right]$ $B=\left[\begin{array}{c} -0.3263\\ -0.0465\\ 0.1575 \end{array}\right]$ RMS: 0.0638 D R^2^: 0.9995	$\left[\begin{array}{c} ZSEQp\\ ZC0p\\ ZC45p \end{array}\right]=A\cdot \left[\begin{array}{c} HSEQp\\ HC0p\\ HC45p \end{array}\right]+B$ $A=\left[\begin{array}{ccc} 0.9274 & -0.0013 & 0.0062\\ 0.0162 & 0.8713 & -0.0512\\ -0.0136 & 0.0026 & 0.8387 \end{array}\right]$ $B=\left[\begin{array}{c} -0.0980\\ 0.0823\\ -0.0880 \end{array}\right]$ RMS: 0.0662 D R^2^: 0.9994

## Data Availability

The data presented in this study are available on reasonable request from the corresponding author. The data are not publicly available because they contain clinical measurements obtained during routine patient care.

## Abbreviations

The following abbreviations are used in this manuscript:

ACD	Anterior Chamber Depth
Anterion	Heidelberg Engineering Anterion
APR	Anterior to posterior corneal curvature ratio
AS-OCT	Anterior segment optical coherence tomography
C0	Astigmatic power vector component in the 0/90 degree meridian
C45	Astigmatic power vector component in the 45/135 degree meridian
CCT	Central corneal thickness
IOL	Intraocular lens
IOLM	Zeiss IOLMaster 700
MMV	Multivariate linear model mapping power vector components
MR	Linear regression model without intercept
MRI	Linear regression model with intercept
RMS	Root mean squared
SD	Standard deviation
SEQ	Spherical equivalent power
SS-OCT	Swept-source optical coherence tomography
TK	Total keratometry
